# Screening and Identification of Multiple Peptides Homologous to the Fusion Glycoprotein Gc of Schmallenberg Virus Able to Inhibit Viral Infection

**DOI:** 10.1155/tbed/1600862

**Published:** 2025-07-18

**Authors:** Carla Zannella, Rinaldo Grazioso, Annalisa Chianese, Valentina Iovane, Biagio Santella, Serena Montagnaro, Giuseppe Greco, Ugo Pagnini, Gianluigi Franci, Anna De Filippis, Giuseppe Iovane, Carla Isernia, Massimiliano Galdiero

**Affiliations:** ^1^Dipartimento di Medicina Sperimentale, Università degli Studi della Campania Luigi Vanvitelli, Naples, Italy; ^2^Dipartimento di Scienze e Tecnologie Ambientali, Biologiche e Farmaceutiche, Università degli Studi della Campania Luigi Vanvitelli, Caserta, Italy; ^3^Dipartimento di Agraria, Università degli Studi di Napoli Federico II, Portici, Italy; ^4^Dipartimento di Medicina, Chirurgia e Odontoiatria “Scuola Medica Salernitana”, Università degli Studi di Salerno, Baronissi, Italy; ^5^Dipartimento di Medicina Veterinaria e Produzioni Animali, Università degli Studi di Napoli Federico II, Naples, Italy

**Keywords:** bunyavirus, fusion membrane proteins, inhibitory peptide, one health, schmallenberg virus, spillover

## Abstract

Arthropod-borne viruses have been responsible for several emerging infections, causing a global issue in both human and veterinary fields. Within the Orthobunyaviruses, a novel and major member is the Schmallenberg virus (SBV) first detected in central Europe in 2011, and soon after was able to spread all over the continent by causing severe infection in ruminants, leading to abortion and congenital malformations. The viral particle is surrounded by a membrane in which two glycoproteins (Gn and Gc) mediate the entry, mainly through the class II fusion protein Gc, but this event requires the presence of Gn. Therefore, Gn and Gc may represent a target for antiviral development. In our study, we evaluated the inhibitory effect mediated by overlapping peptides designed on the amino acid sequences of Gc and Gn and spanning their entire length. A brute analysis of both glycoproteins was performed to explore the inhibitory effect of such peptides against SBV infection. Five out of 63 Gc peptides at a concentration of 100 μM reached 50% of inhibition and, interestingly, they are mainly distributed near the C-terminal domain. None of the 20 Gn peptides inhibited the infection, and no peptide toxicity was observed. Our findings could identify new putative domains, located at the C-terminal of Gc, in the process of SBV penetration; therefore, these results are relevant to the potential development of novel therapeutic agents for the treatment of SBV infections and could serve as a model for many human pathogens belonging to the same family.

## 1. Introduction

Enveloped viruses can cause infection after entering susceptible cells by fusing the virus membrane with a host cell membrane [1]. This key fusion event between opposing membranes allows the delivery of the viral genome within the cell cytoplasm to initiate infection, paving the way for viral replication. Membrane fusion is mediated by virally encoded transmembrane proteins known as viral fusion proteins which, following an appropriate stimulus, such as pH acidification, undergo conformational modifications [2]. Thus, the interactions with the target membrane, through a hydrophobic fusion peptide located in the viral fusion protein, accomplish the membrane fusion reaction [3]. Based on structural criteria, three types of viral fusion proteins, each exhibiting distinct structure folds, have been described so far. Within class I viral fusion protein, the fusion subunit is mainly composed of *α*-helical domains, with N- and C-helical heptad repeats, which are structured to form a six-helix bundle containing a trimer-of-hairpins in the postfusion conformation and an N-terminal fusion peptide. Class II fusion proteins consist of *β*-strands and *β*-sheets, and the fusion loop(s) is located at the tip of an extended *β*-sheet domain, while class III fusion proteins are characterized by *α*-helical and *β*-sheet regions, with fusion loops very similar to class II [1]. Molecular details on virus entry mechanism are of importance to our overall understanding of viral infection pathways, hence targeting the entry process is a promising strategy to design antiviral therapies [4].

Schmallenberg virus (SBV), an orthobunyavirus belonging to the *Peribunyaviridae* family, was initially identified in 2011 in infected cows on a farm in the area close to the town of Schmallenberg in Germany (from which it takes the name) [5] and has since spread causing several epidemics in farms all over Europe [6]. SBV is an arthropod-borne virus where midges of the *Culicoides* species are the way of transmission to ruminants, and generally causes mild symptoms in adult animals. Whereas infection of pregnant livestock can be deleterious and may lead to severe congenital malformations, miscarriages, or stillbirths [7]. SBV belongs to the order *Bunyavirales*, among which we find several zoonotic viruses of great interest for the high mortality rates they can cause in humans, such as the Crimean-Congo hemorrhagic fever virus (CCHFV), La Crosse virus (LACV), Rift Valley fever virus (RVFV), Ngari virus causing an hemorrhagic fever, or Oropouche virus [8]. SBV has been classified as a biosafety level 2 pathogen since it is not known to be pathogenic to humans [7]. Therefore, not being a threat to public health, this virus can be exploited as an experimental model of other zoonotic orthobunyaviruses able to cause disease outbreaks in humans since they require a higher level of containment.

Similarly to other orthobunyaviruses, SBV possesses a tripartite genome composed of three RNA segments of negative polarity, designated small (S), medium (M), and large (L) segments, encoding four structural proteins (N, nucleoprotein; Gn and Gc, glycoproteins; L, RNA-dependent RNA polymerase) and two nonstructural proteins (NSm and NSs). The M segment codes for the glycoprotein precursor (GPC; in the following order Gn-NSm-Gc) that requires a cleavage for producing the mature viral glycoproteins Gn and Gc and a nonstructural protein, NSm. Gn (32–35 kDa) and Gc (100–110 kDa) form the spikes on the viral envelope that perform fundamental functions in viral attachment and penetration into susceptible cells. Both surface glycoproteins are type I integral transmembrane proteins with N-linked glycosylated sites and rich in cysteine residues. Gn and Gc form a heterodimer in the endoplasmic reticulum (ER) and are transported to the Golgi apparatus where virion assembly occurs. The glycoprotein predicted to contain a fusion peptide in the C-terminal core region is the Gc and is considered a class II membrane fusion protein [9]. Limited information is currently available on the structure of the orthobunyavirus glycoproteins: the crystal structure of the N-terminal half of SBV Gc has been resolved (Protein Data Bank, PDB 6H3S) [10] and, recently, also the fusion core has been described (PDB 7A56); on the contrary, no data on Gn structure have yet been reported. The N-terminal half of SBV Gc is the most variable and presents an elongated domain with a protruding *α*-helical head domain positioned on a stalk region composed of two *β*-sheet subdomains [11]. The N-terminal half of Gc is the most exposed and it is, therefore, involved in antibody recognition. Its deletion results in a mutant virus still able to replicate, suggesting that the fusion event is driven by other functional domains. Hellert et al. reported the structure of almost all the remaining parts of Gc which, being more conserved, is reminiscent of other pH-dependent class II fusion proteins. Gn and Gc decorating the surface of the virion have a role in binding to cell surface molecules (such as heparan sulfate proteoglycans) in the initial step of SBV infection. This initial attachment is followed by the uptake of viral particles into the endocytic cellular machinery until they reach the late-endosomal vesicles. At this stage, nucleocapsids enter the cytosol following endosomal acidification due to the influx of ions, such as potassium and calcium, which provide the trigger for the conformational changes needed to induce membrane fusion and the consequent uncoating.

Although the precise and complete mechanism by which SBV and other viruses gain full access into cells remains partially elusive and, also considering that the three classes of viral fusion proteins display distinct structural folds, a common mechanism of membrane fusion and supramolecular protein–protein interactions is surely shared. Furthermore, the interactions leading to the membrane fusion are also involved in several interactions of the fusogenic protein with the lipid interface of the opposing membrane, therefore peptides interfering with such interfaces may serve as promising leads in drug discovery and useful tools to further dissect the molecular mechanism of membrane fusion. An established example can be peptides that mimic the C-terminal repeat coil regions of the human immunodeficiency virus type 1 (HIV-1) gp41 and has been demonstrated to be powerful inhibitors for the HIV entry process. The first Food and Drug Administration (FDA) approved fusion inhibitor is Enfuvirtide, a peptide that mimics one of the two helices of the postfusion six-helix bundle of the HIV-1 gp41 protein and effectively inhibits conformational change during fusion, thus preventing viral infection [12]. The success achieved with Enfuvirtide has led to research aimed at designing inhibitors for class II and class III envelope fusion proteins. Studies have shown that several hydrophobic peptides derived from the class II envelope proteins of DENV and WNV have strong inhibitory activities [13]. Moreover, also peptides derived from the class III Herpes simplex virus type 1 (HSV-1) gB exhibited a consistent antiviral activity [14, 15].

Recently, multiple studies have analyzed viral glycoproteins using a generalized peptide-based approach to identify key functional areas. The precise mechanism explaining how some synthetic peptides inhibit membrane fusion is not yet fully elucidated. Many regions of glycoproteins are situated on their surfaces, indicating potential interactions with other proteins and lipids in the cell membrane. In addition, certain peptides may become exposed on the exterior of the glycoprotein due to conformational rearrangements during the fusion process. Many peptides with antiviral potential may be underscored by a surface analysis of the protein; therefore, a brute force analysis able to identify peptide entry inhibitors may aid in the search for viral fusion glycoproteins domains [16, 17].

Here, we employed a peptide scanning inhibition strategy to effectively identify key functional domains, paving the way for the development of promising lead compounds to combat infections. We designed a series of overlapping peptides that thoroughly represent the complete sequences of the SBV Gn and Gc ectodomains. Our screening process evaluated 20 Gn peptides and 63 Gc peptides for their potential to inhibit SBV infection in BHK-21 cells, starting from a concentration of 100 μM. Subsequently, their mechanism of action has been investigated as well as their secondary structures, evidencing that two Gc peptides were the more active and could be used as entry inhibitors against SBV infection.

## 2. Materials and Methods

### 2.1. Peptide Synthesis

Peptides were acquired from Shanghai Ruifu Chemical Co., Ltd (Shanghai, China). All purified peptides were obtained with high yields (50%–60%) and have a purity above 90%.

### 2.2. Cells and Viruses

Baby Hamster Kidney cells (BHK-21) (ATCC CCL-10, Manassas, VA, USA), Madin–Darby bovine kidney (MDBK, ATCC CCL-22), and Vero/hSLAM cells (ECACC 04091501, Porton Down, United Kingdom) were cultivated in Dulbecco's modified Eagle's medium (DMEM, Microtech, Naples, Italy) supplemented with 10% fetal bovine serum (FBS) (Microtech). Viruses were kindly provided by Professor Giuseppe Iovane (University Federico II, Naples, Italy). Cells were infected with a multiplicity of infection (MOI) of 0.01, and we assessed viral titers through plaque assays.

### 2.3. Plaque Titration

SBV titration was performed on BHK-21 cells. Briefly, BHK-21 cell monolayers in 12-well cell culture plates were infected with SBV dilutions. After 1 h of incubation, viral inocula were removed and a mixture of DMEM 10% FBS and 3% carboxymethyl cellulose (CMC) was added to all wells. After incubation for 72 h, cells were fixed with 4% formaldehyde solution and stained with 0,5% crystal violet (Sigma-Aldrich, St. Louis, MO, USA). Plaques were scored and stock virus titration was calculated as plaque forming unit (PFU)/ml.

### 2.4. Cotreatment Assay

Peptides were dissolved in water. To assess the impact of these peptides on inhibiting SBV infectivity, a very general assay (cotreatment) was performed by treating cells (5 × 10^5^ cells/well) with peptides at 200, 100, 50, 25, 12.5, 6.25, and 1 μM and incubating them with the viral inoculum (MOI of 0.01) for 2 h at 37°C. To effectively inactivate nonpenetrated viruses, we treated cells with citrate buffer (pH 3.0) and then cultured BHK-21 for 72 h at 37°C/5% CO_2_ in DMEM enriched with 10% FBS and CMC. The monolayers were finally fixed and stained, and the plaque number was scored. The percentage of inhibition was calculated in comparison to the control group (cell infected and treated with no peptide).

### 2.5. Time of Addition Assays

Peptide concentrations are the same as used in the initial cotreatment assay. Virus pretreatment assay: peptides were added to the virus (MOI of 0.1) for 1 h at 37°C. Following the incubation period, the mixture of virus and peptide was diluted on cells for 2 h: this process allowed the peptides to achieve a nonactive concentration while ensuring that the virus remained at an optimal final MOI of 0.01. Then, the cells underwent a 72-h incubation with CMC. Cell pretreatment: Precooled cells were treated with the peptides for 1 h, followed by the incubation of the viral inoculum (MOI of 0.01) for 2 hr at 37°C. At the end, cells were overlaid with CMC for 72 hr at 37°C. Posttreatment: Cells were treated with peptide following the viral infection (MOI of 0.01 pfu/mL) for 2 h at 37°C; then, CMC was added for 72 h at 37°C. For each treatment, the IC50, which is the peptide concentration resulting in a 50% reduction of SBV infectivity, was calculated.

### 2.6. Temperature Shift Assays

Two additional assays were performed. Peptide concentrations are the same as those used in the other treatments. Attachment assay: Cells were infected with the virus (MOI 0.01) and simultaneously each peptide was inoculated for 2 h at 4°C; then, cells were left in CMC for 72 h at 37°C. Entry assay: BHK-21 monolayer was infected with SBV (MOI 0.01) for 2 h at 4°C, followed by the treatment with each peptide for 1 h at 37°C; finally, CMC was added on cells. Similarly to other assays, at the end, viral plaques were observed.

### 2.7. Toxicity

Peptide cytotoxicity was measured by 3- (4, 5-dimethylthiazol-2-yl)-2, 5-diphenyltetrazolium bromide (MTT) assay according to manufacturer's instructions (Sigma-Aldrich, St. Louis, Missouri, United States). BHK-21 cells (4 × 10^4^ cells/well) were treated with peptides at different concentrations ranging from 1 µM to 200 and, after 24 h, 100 μL of the MTT solution (5 mg/mL) was incubated for 3 h at 37°C. Then, we used DMSO (Sigma Aldrich) to dissolve tetrazolium salts under agitation. Cytotoxicity was analyzed at 570 nm, and cell viability was measured against control cells—those that received no treatment. At the end, the 50% cytotoxic concentration (CC_50_) was determined using GraphPad Prism software (GraphPad Software, San Diego, CA, USA).

### 2.8. Real-Time PCR

Cotreatment assay was performed as indicated above. After 48 h, RNA was extracted by using TRIzol reagent (Thermo Fisher Scientific, Waltham, MA, United States), and its absorbance was quantified. RNA was reverse transcribed and the resulting cDNA was amplified through a quantitative polymerase chain reaction. SBV M gene expression was evaluated. Relative target threshold cycle (Ct) values were normalized using the housekeeping gene Glyceraldehyde 3-phosphate dehydrogenase (GAPDH). Primers used in this study are M gene forward, TCAATTCAGCAAGTAACATACAATGG; M gene reverse, CGTGGTCTGTCTTGGTTGATG; GAPDH gene forward, CCTTTCATTGAGCTCCAT; GAPDH gene reverse, CGTACATGGGAGCGTC, (Eurofins Scientific, Luxembourg). Analysis was conducted by using the 2^-ΔΔCt^ method.

### 2.9. Circular Dichroism

Circular dichroism (CD) measurements were collected using a JASCO J-815 CD spectropolarimeter (Tokyo, Japan) equipped with Peltier temperature control. Each sample was prepared in water by adding 50 µM of TCEP (tris (2-carboxyethyl) phosphine), and the pH was adjusted from ~4 to 7 with NaOH and HCl 0.01M solutions; the peptide concentration was 15 µM. Data were collected at 298K using a quartz cuvette with a 1 cm path length in the 190−260 nm wavelength range with 1 nm data pitch and 50 nm/min scanning speed and normalized to the reference spectra to remove the background contribution of the buffer. The *θ* values at 222 nm and at 195 nm were followed to monitor the *α*-helix and *β*-sheets percentages that were calculated by the server BeStSel. Data were fitted using the program GraphPad Prism 7.0.

### 2.10. Statistical Analysis

All the described tests were performed in triplicate and expressed as mean ± Standard Deviation (SD) calculated by GraphPad Prism, version 8.0.1. One-way ANOVA followed Dunnett's multiple comparisons test was used; a value of *p* ≤ 0.05 was considered significant.

## 3. Results

**Figure 1:**
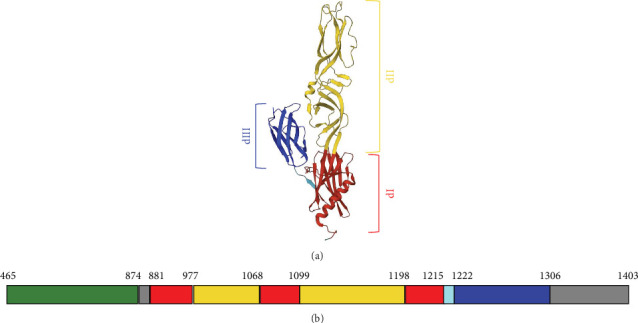
SBV Gc. (A) At the top of the panel, Gc fusion domains in post-fusion conformation are indicated with domains I, II, and III colored in red, yellow, and blue, respectively. DI-DIII linker is ciano-colored. (B) The bottom of the panel shows a linear diagram of Gc sequence color-coded to match the structure above and labeled to indicate the boundary of each region with respect to the entire envelope polyprotein (UNIPROT code H2AM12). The sequence refers to the deposited structure present on PDB (code 7A56), while the green color indicates Gc head/stalk domains (PDB code 6H3S), and the gray color refers to the non-predicted domains.

Comparing the structure of this protein to others structures already described, that is the flaviviruses dengue virus (DENV) and tick-borne encephalitis virus (TBEV) E proteins, the togaviruses Semliki Forest virus (SFV) and Rubella virus E1 proteins, the bunyaviruses RVFV, and Heartland virus Gc glycoproteins [18–20], SBV Gc is similarly subdivided into three domains rich in *β*-strands (Figure 1).

Figure 1 shows the three-fold structure of Gc from residue 881 to 1306. Domain III is the central *β*-sandwich extending from residue 1222 to 1306, and it is flanked by domain I (881–977; 1068–1099; 1198–1215) on one side and domain II (974–1068; 1099–1198) on the other side connecting to the transmembrane region. Domain II has an elongated shape and contains the fusion loop able to insert into the target cellular membrane, meanwhile, domain III has a typical IgC-like structure with a seven-stranded *β*-sandwich.

**Figure 2:**
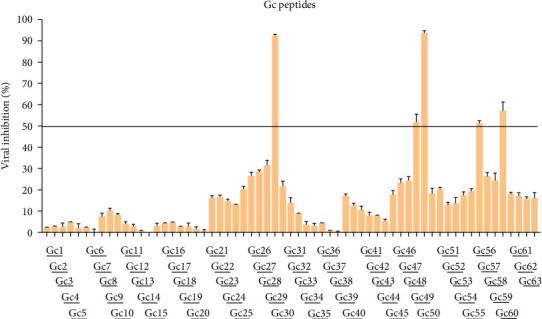
Inhibitory activity of SBV Gc peptides. Peptides were incubated at the same time of the infection at a concentration of 100 μM. The antiviral effect was expressed as a percentage of viral inhibition. A threshold line was established at 50% inhibition to highlight significant results.

To pinpoint critical functional regions within SBV membrane glycoproteins, we developed two peptide libraries derived from the sequences of Gc and Gn: (i) a series of 25-mer peptides spanning from residues 18 to 303 of Gn, starting just after the signal peptide of the envelope M polyprotein (Uniprot H2AM12); (ii) 25 amino acids-long peptides derived from the residues 453 to 1403 of Gc. Both the Gc and Gn libraries comprised peptides that overlapped by 10 amino acids (Table S1), while we excluded the region encoding for the non-structural protein M from our analysis (from residue 328 to 452 of the M polyprotein). We evaluated the inhibitory activity of peptides using a cotreatment assay, by incubating both the virus and peptides simultaneously on cells. A peptide was considered worthy of further investigation if it achieved at least 50% inhibition of infection at 100 μM (Figure 2).

**Figure 3:**
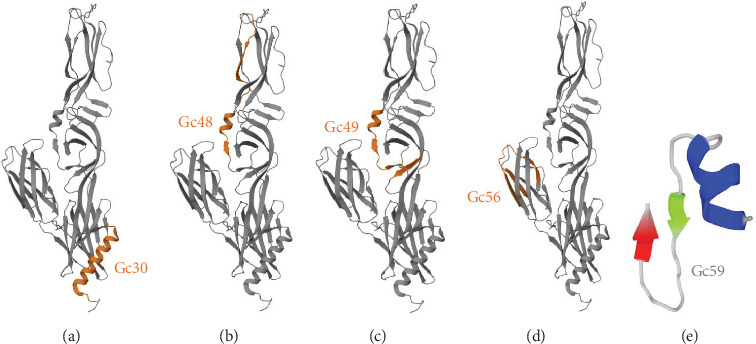
Peptide distribution in Gc monomer. (A) Gc30 (888–912); (B) Gc48 (1158–1182); (C) Gc49 (1173–1197); (D) Gc56 (1278–1302); (E) Gc59 (1323–1347) is not present in the annotated Gc structure and has been predicted by PEP-FOLD3 software. Neither the stem nor the transmembrane region is noted in the PDB structure.

The criteria for classifying peptides as potential inhibitors were established based on the typically elevated concentrations necessary to achieve effective antiviral activity with peptides derived from Class II viral fusion proteins. Previous studies indicate that peptide concentrations between 10 and over 100 μM are required to inhibit the viral infectivity of viruses with Class II glycoproteins [21, 22]. Out of the 63 peptides identified in the Gc library, five exhibited an inhibition activity greater than 50% (Figure 2), while none of the Gn peptides reached the threshold (Figure S1). Interestingly, the 5 Gc peptides showed inhibitory activity clustered in the core region within the Gc.  Figure 3 illustrates the precise positions of the active peptides in the Gc, which we have categorized into three distinct regions for clearer understanding: (i) Gc30 corresponded to the *α*-helix within domain I; (ii) Gc48 and Gc49 were overlapping peptides located in domain II; (iii) Gc56 and Gc59 were positioned in domain III within the barrel-stave structure. However, since Gc59 sequence was not reported in the deposited Gc structure (881 to 1306), we predicted its structure by PEP-FOLD3 software.

Once a subset of peptides endowed with antiviral activity was selected based on this high-throughput screening, we used the selected peptides to better investigate their mechanism of action by analyzing them via different assays, namely cotreatment, cell pretreatment, virus pretreatment, attachment, and entry assays, and posttreatment. Different noncytotoxic concentrations of peptides (ranging from 100 to 1 μM) were used and results are indicated in Table 1 with the corresponding CC_50_, IC_50_, and selectivity index (SI).

First, each peptide and the virus were mixed and directly incubated on the BHK-21 cell monolayer in the experiment indicated as a cotreatment assay. After 2 h to favor virus attachment and entry, the mixture peptide/virus was eliminated, and cells were covered with CMC for 72 h. Finally, viral plaques were counted to measure the ability of peptides to inhibit the viral infection. Among the five peptides selected from the previous screening, two peptides, specifically Gc30 and Gc49, were the most active: Gc30 and Gc49 exhibited an IC50 at 12.5 and 10 μM, respectively. Further, we evaluated if the peptides could directly interfere with the viral surface or indirectly interact with the target cells prior or after the infection. Therefore, we performed the virus pretreatment assay to elucidate if peptides could interact directly with SBV particles, preventing subsequent stages of infection. In brief, instead of incubating the virus and peptides directly on cells, peptides were first added to viral particles for 1 h at 37°C; then, the mixture of peptide/virus was diluted on BHK-21 cells and incubated for a *supplementary* hour. After that, cells were overlaid with CMC for 72 h, and infection was quantified by plaque counting. All the peptides inhibited SBV infection, as evidenced by the higher SI values. In detail, Gc30 and Gc49 were again the most active, with an IC_50_ at 20 and 10 μM, respectively. Conversely, peptides were not active in cell pretreatment and posttreatment assays (Table 1). Our results indicated that the Gc active peptides could inhibit the early phases of infection by acting mainly on the viral surface. Different mechanisms could explain how these peptides exert their inhibitory effect. They could cause interference with the correct organization of the envelope's lipid bilayer, interact with the viral glycoproteins, or simply constitute a steric hindrance by interfering with the attachment and fusion phases of infection [23, 24].

At this point, it was necessary to understand when the peptides exerted their virucidal role during the adsorption, viral attachment, and/or entry steps. To elucidate the precise moment in which peptides could interfere with the SBV lifecycle, two different temperature shift assays were carried out. These assays depend on the temperatures required for the two initial steps on the virus lifecycle (4°C for the attachment and 37°C for the entry). Therefore, in the attachment assay, BHK-21 cells were treated with peptide and simultaneously infected by SBV at 4°C, where the virus only attached to cells without penetrating them. Peptides exhibited only a reduced effect compared to their activity in virus pretreatment (Table 1), while their activity has been mainly referred to the entry step (Table 1). Here, cells were first infected by SBV at 4°C to prevent the virus penetration; then, cells were treated with peptide and shifted at 37°C, where the viral entry can occur. Table 1 indicated that peptides, principally Gc30 and Gc49, reduced the viral entry with an IC50 at 20 μM. This observation suggested that peptides could prevent an event immediately following the viral adsorption, i.e., the conformational change occurring during the fusion stage.

**Figure 4:**
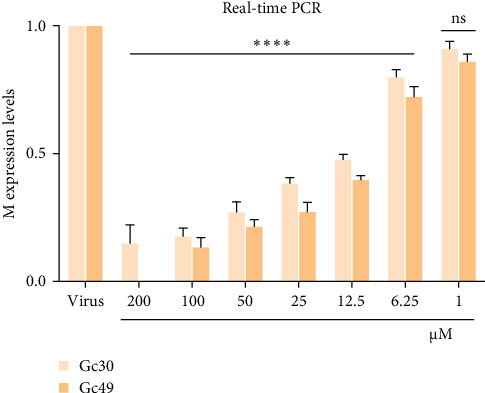
Real-time PCR was performed to evaluate the effect of Gc30 and Gc49 on M gene expression in co-treatment assay. After a 48-h incubation period, RNA was extracted, cDNA was produced, and subsequently amplified through real-time PCR. Virus indicates the infected cells. *⁣*^*∗∗∗∗*^*p* < 0.0001; ns, not significant.

The antiviral activity of Gc30 and Gc49 has been further validated through the analysis of the M gene expression level. SBV has a tripartite RNA genome where the M segment encodes the two viral surface glycoproteins, Gn and Gc. BHK-21 cells were infected with the virus and simultaneously treated with each peptide in the range of concentration from 200 to 1 μM (Figure 4). After 48 h postinfection, RNA was extracted, reverse transcribed in cDNA, and real-time PCR was performed. Figure 4 showed a dose-dependent inhibition of the viral M gene as peptide concentration increased.

**Figure 5:**
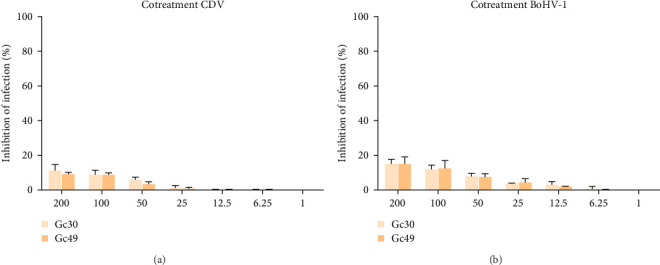
Co-treatment assay with (A) CDV and (B) BoHV-1, to exclude any antiviral effect of Gc30 and Gc49 against viruses with diverse classes of membrane fusion proteins.

To evaluate the potential toxicity of the peptides on BHK-21 cells, we treated monolayers with each peptide at concentrations of 10, 50, 150, and 200 μM for 24 h, and then MTT test was performed. There was no significant difference in the viability of untreated cells compared to those treated with the peptides (Table 1, CC50). This indicates that the peptides tested do not exhibit cytotoxic effects at the concentrations used, reinforcing their potential safety for further research. Another point we excluded was that SBV peptides could exert an antiviral effect also against other types of viruses embedded with to different classes of membrane fusion proteins. Therefore, we tested the activity of Gc30 and Gc49 against two animal viruses exposed class I fusion protein, i.e., the canine distemper virus (CDV), and class III fusion protein, i.e., the bovine herpesvirus-1 (BoHV-1). We performed a cotreatment assay and results in Figure 5 indicate that no peptide designed on SBV Gc fusion protein was able to block the infection caused neither by CDV nor BoHV-1.

**Figure 6:**
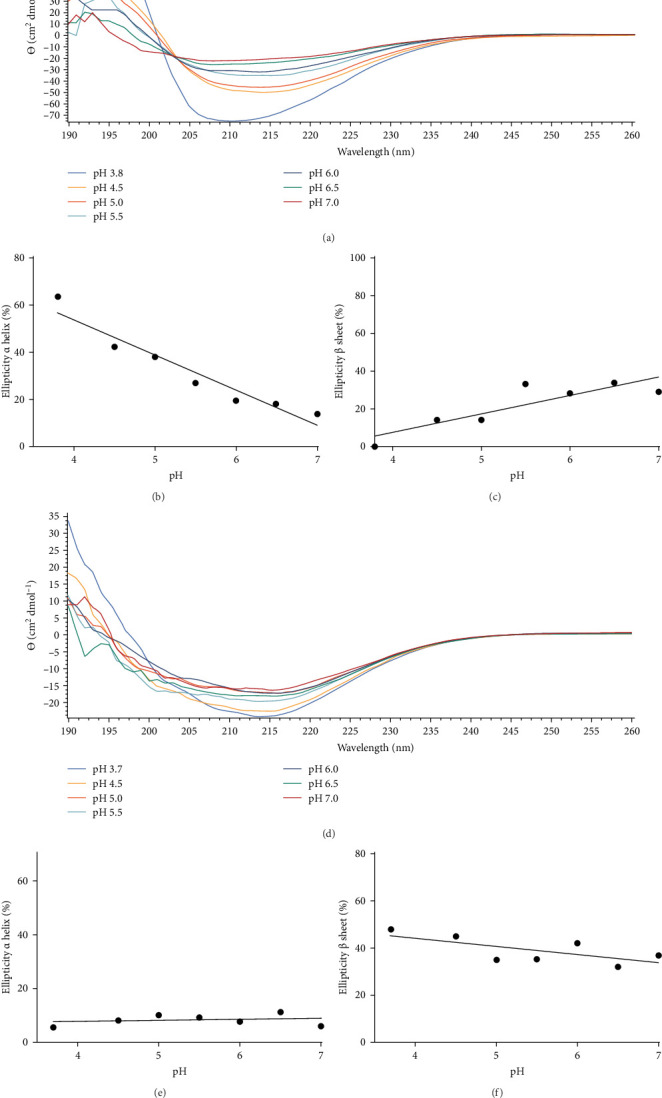
CD spectra of Gc30 (A) and Gc49 (D) were reported as a function of pH. The *α*-helix and *β*-sheet contents for peptide Gc30 (B, C) and Gc49 (E, F) were calculated by the server BeStSel.

We finally explored the structure–activity relationship between the peptides and their antiviral activity. CD spectra, reported as a function of pH, and calculated percentages trend of secondary structure content, for all the five active peptides, are reported in Figure 6 and Figure S2.

It can be noted that Gc30 showed a major change of conformation as a function of pH, while Gc48, Gc49, Gc56, and Gc59, were only slightly affected by the pH increment.

In particular, Gc30 exhibited a negative band centered at 210 nm at each pH value (Figure 6A); the band, which was wide at pH 3.8, resulted attenuated already at pH of 4.5 and further decreased at neutral pH. Accordingly, the calculated percentage of *α* -helix decreased from 64% to 14% as the pH was raised (Figure 6B); furthermore, a simultaneous increase of *β*-strand from 0% to 35% was observed (Figure 6C).

As far as the behavior of Gc49 raising the pH from 4 to 7 (Figure 6D), CD spectra evidenced a decrease in the *β*-sheet content of about 10% (from 48% to 38%, Figure 6F) but not of the small *α* helix percentage that remained almost unchanged (ca 10%) (Figures 6E).

Conversely, Gc48, Gc56, and Gc59 (Figure S2) showed a very low level of *α*-helix at acidic pH with percentages that vary from 2% for Gc59 to ca 15% for Gc48. With the increase of pH only Gc56 and Gc59 exhibited a slight, about 5%, increment in the helix percentages. The content of the *β*-strand resulted in about 45% for Gc56 and Gc59 and linearly diminished to 40% at pH 7. Gc48 showed a very constant trend with the pH, maintaining the content of *β*-strand and *α*-helix at about 35%–40% and 15%, respectively, for all the ranges examined.

## 4. Discussion

The COVID-19 Disease 2019 (COVID-19) pandemic, caused by the severe acute respiratory syndrome coronavirus 2 (SARS-CoV-2), profoundly impacted globally, raising awareness and intensifying concerns about highly transmissible respiratory infections. However, beyond coronaviruses, other viral pathogens continue to pose serious threats to both human and animal health. Among these, bunyaviruses have gained increasing recognition due to their potential to cause widespread outbreaks. The World Health Organization (WHO) has included three bunyaviruses—RVFV, CCHFV, and severe fever with thrombocytopenia syndrome virus (SFTSV)—in its Blueprint list of priority pathogens with epidemic potential. Despite this classification, there are currently no approved antiviral treatments or vaccines for human use, although veterinary vaccines are under development for certain bunyaviruses [25].

A major obstacle to developing effective countermeasures lies in the limited understanding of bunyavirus glycoprotein structures and their functional roles in viral replication. Structural and functional characterization of viral glycoproteins is crucial for identifying potential therapeutic targets. To date, only a small number of studies have focused on nucleoside analogs capable of inhibiting bunyavirus replication by targeting RNA-dependent RNA polymerase (RdRp). Ribavirin, a broad-spectrum antiviral first introduced in the 1970s, [26, 27] has demonstrated efficacy in reducing mortality in rodent models infected with CCHFV, [28] SFTSV, [29] and hantavirus[30]. Another nucleoside analog, favipiravir, originally developed for influenza treatment, [31] has shown promising broad-spectrum antiviral activity against bunyavirus infections[32]. Notably, in a clinical trial conducted in 2016, favipiravir significantly reduced the case fatality rate in patients infected with SFTSV, particularly in individuals aged 70 years or younger[33].

Viral glycoproteins play a critical role in mediating membrane fusion during viral entry, undergoing large conformational changes that facilitate the merging of the viral and host cell membranes [34]. These structural rearrangements make viral glycoproteins an attractive target for the development of fusion inhibitors [35]. Molecules that bind to an intermediate state of the fusion protein, preventing its transition to the postfusion conformation, could serve as highly effective and virus-specific inhibitors. Previous studies have demonstrated the potential of peptide inhibitors targeting class II fusion proteins. Peptides derived from the stem region of DENV, West Nile virus (WNV), and Japanese encephalitis virus (JEV) [36] have exhibited inhibitory effects on viral entry, highlighting the feasibility of this approach. Similarly, peptides modeled on the fusion stem of RVFV have shown cross-inhibitory activity against multiple viruses, including hantaviruses, Ebola virus (EBOV), and vesicular stomatitis virus (VSV) [37].

Despite significant progress in structural virology, the structural characterization of bunyavirus glycoproteins remains incomplete. To date, only the structure of the SBV Gc protein has been determined (PDB codes 6H3S and 7A56), while the structure of the Gn protein remains unknown. Gc, a class II fusion protein, is primarily responsible for mediating the fusion of viral and host cell membranes, whereas Gn serves as a transporter, ensuring proper folding and localization within the Golgi apparatus. In the present study, a library of synthetic peptides targeting SBV glycoproteins was analyzed to evaluate their antiviral efficacy *in vitro*. The results demonstrated that peptides derived from functional domains of the SBV Gc protein were capable of inhibiting viral infection (Figure 2 and Table 1), suggesting their potential as antiviral agents.

Among the identified peptides, Gc30 and Gc49 exhibited the strongest anti-SBV activity. We also tested their antiviral potential against two other animal viruses with different classes of membrane fusion proteins on their surface. As models, we selected CDV, which uses the class I F protein for fusion, [38], and BoHV-1, which relies on class III glycoprotein B for its membrane fusion process [39]. Neither Gc30 nor Gc49 showed any effect against CDV or BoHV-1 infection (Figure 5), demonstrating that their antiviral activity is specific to SBV inhibition. Structural analysis revealed that Gc30, located in domain I of the Gc protein (Figure 3A), adopts an *α*-helical conformation at acidic pH (Figure 6A–C), a property that may contribute to its interaction with viral membranes and inhibition of fusion [40, 41]. Further experiments are needed to confirm whether the antiviral specificity of Gc30 is indeed linked to its *α*-helical structure and its ability to permeabilize liposome membranes. In contrast, Gc49, positioned within the hinge region between domains I and II (Figure 3C) and with a *β*-sheet conformation (Figure 6D–F), appears to interfere with the conformational flexibility necessary for the transition from pre-fusion to post-fusion states.

The pH-dependent nature of these peptides suggests that they may act by disrupting the pH-triggered conformational changes essential for viral entry [42, 43]. Given that bunyavirus fusion occurs within a narrow pH range, targeting these acid-induced transitions could represent an effective antiviral strategy. Endosomal acidification is a critical step in viral entry, as it triggers the conformational changes required for membrane fusion. Several studies have explored inhibitors that interfere with endosomal acidification, with compounds such as chloroquine being shown to increase endosomal pH and inhibit the replication of pH-dependent viruses, including SARS-CoV-2 [44, 45]. Bunyaviruses also rely on endosomal acidification for entry, with fusion occurring at pH values ranging from 5.4 to 6.0, depending on the specific virus. The presence of histidine, glutamate, and aspartate residues within viral glycoproteins allows them to function as pH sensors, [46, 47] facilitating protonation and subsequent conformational rearrangements. The identified SBV-derived peptides, Gc30 and Gc49, contain several of these pH-sensitive residues, which may contribute to their ability to interfere with the fusion process. Further investigations are needed to confirm their role in inhibiting viral entry by stabilizing glycoproteins in an inactive state.

A potential strategy to enhance the antiviral efficacy of these peptides is conjugation with cholesterol [48–50]. Cholesterol-peptide conjugates have been shown to improve membrane targeting and increase the stability of fusion inhibitors. This approach has been widely used to enhance the bioavailability of antiviral peptides, facilitating their incorporation into lipid rafts and prolonging their half-life *in vivo*. By promoting more efficient membrane interaction, cholesterol conjugation could further improve the therapeutic potential of Gc-derived peptides.

In addition to targeting the fusion protein itself, the conserved nature of fusion loops within domain II of SBV Gc presents another promising antiviral target. These loops, which include the bc, cd, and ij loops, consist primarily of non-polar residues and play a crucial role in membrane insertion during the fusion process. Previous studies have suggested that similar regions in hantavirus Gc are essential for transitioning to the post-fusion conformation [51, 52]. The identification of peptides capable of binding to these fusion loops, such as Gc48, could offer an alternative strategy for inhibiting viral entry. Given the high degree of conservation of these regions among orthobunyaviruses (Figure S3), inhibitors targeting these sites may exhibit broad-spectrum antiviral activity with a lower likelihood of resistance development.

The findings of this study provide new insights into the structural and functional properties of bunyavirus glycoproteins while highlighting the potential of peptide-based inhibitors as antiviral agents. By interfering with key steps in the viral entry process, these peptides could serve as the basis for novel therapeutic interventions against bunyavirus infections. Future studies should focus on optimizing peptide structures, exploring their mechanisms of action, and evaluating their efficacy in vivo. Additionally, the combination of peptide inhibitors with established antiviral drugs may enhance treatment outcomes and offer a comprehensive strategy for managing bunyavirus infections.

## 5. Conclusions

Hopefully, this inhibitory strategy, based on the modeling of peptides on class II fusion proteins, will contribute to the future development of therapeutic strategies not only for bunyaviruses like SBV but also for other types of viruses, following the same approach previously applied to class I glycoproteins. Analyzing the differential behavior between highly pathogenic virus and nonpathogenic surrogates (such as SBV) can provide valuable insights into the cellular or viral factors that contribute to the pathogenic nature of viruses in humans. Looking ahead, our future research will focus on testing SBV Gc-derived peptides against other members of the *Orthobunyavirus* genus, such as LACV, Cache Valley virus (CVV), and Oropouche virus (OROV), as well as exploring their efficacy against other virus genera like Hantaviruses and Phleboviruses. This broader analysis could help identify common mechanisms and therapeutic targets for a wider range of viral pathogens, advancing the development of antiviral treatments for these emerging and potentially dangerous viruses.

## Figures and Tables

**Table 1:** Active peptide cytotoxicity and antiviral assays.

Peptide	Sequence	Position	CC50 (*µ*M)	Antiviral treatment	IC50 (*µ*M)	SI
Gc30	NHPDIENYIAALQSDIANDLTMHYF	888–912	150	Cotreatment	12.5	12
				Cell pretreatment	/	/
				Virus pretreatment	20	7.5
				Attachment	50	3
				Entry	20	7.5
				Posttreatment	/	/

Gc48	FNRKDVILRRCFDNSYQSCLLLEQD	1158–1182	200	Cotreatment	100	2
				Cell pretreatment	/	/
				Virus pretreatment	50	4
				Attachment	100	2
				Entry	50	4
				Posttreatment	/	/

Gc49	YQSCLLLEQDNTLTIASTSHMEVHK	1173–1197	200	Cotreatment	10	20
				Cell pretreatment	/	/
				Virus pretreatment	10	20
				Attachment	25	8
				Entry	20	10
				Posttreatment	/	/

Gc56	YSIKLNCPLATETVSVSVCSASAYT	1278–1302	200	Cotreatment	100	2
				Cell pretreatment	/	/
				Virus pretreatment	40	5
				Attachment	50	4
				Entry	40	5
				Posttreatment	/	/

Gc59	YIEQHDKKCSTWLCRVYKEG	1323–1347	200	Cotreatment	50	4
				Cell pretreatment	/	/
				Virus pretreatment	40	5
				Attachment	55	3.6
				Entry	35	5.7
				Posttreatment	/	/

*Note:* Gc peptides were analyzed in six different treatments, namely cotreatment, cell pretreatment, virus pretreatment, attachment, entry, and posttreatment. CC_50_: 50% cytotoxic activity; IC_50_: 50% inhibitory concentration; SI: selectivity index (CC_50_ value/IC_50_ value). /: not registered activity.

## Data Availability

The data that support the findings of this study are available from the corresponding author upon reasonable request.

## Supporting Information

Supporting Information Figure S1. Inhibitory activity of SBV Gn peptides. Peptides were incubated simultaneously to the infection at the concentration of 100 μM. The relative activity was normalized and reported as % of inhibition. Figure S2. CD spectra of Gc48 (A), Gc56 (D), and Gc59 (G) reported as function of pH. The α-helix and β-sheet contents for peptide Gc48 (B, C), Gc56 (E, F), and Gc59 (H, I) were calculated by the server BeStSel. Figure S3. Alignment of bunyavirus fusion loops. Orthobunyaviruses showed high sequence identity (red-colored residues) [52]; on the contrary, bd, cd, and ij loops differ considerably among other genera, such as Hantavirus and Phlebovirus [51]. SBV: Schmallenberg virus; LACV: La Crosse virus; CVV: Cache Valley virus; OROV: Oropouche virus; HTNV: Hantaan virus; RVFV: Rift Valley fever virus. Table S1. Gn and Gc libraries with peptide position in M segment, names and sequences. The five active peptides are yellow-colored.
