# Supplementary material for: Screening and Identification of Multiple Peptides Homologous to the Fusion Glycoprotein Gc of Schmallenberg Virus Able to Inhibit Viral Infection
Source: Transbound Emerg Dis. 2025 Jul 18;2025:1600862. doi: 10.1155/tbed/1600862 (PMC12297138; doi:10.1155/tbed/1600862)
Supplement: Supporting Information — Figure S1. Inhibitory activity of SBV Gn peptides. Peptides were incubated simultaneously to the infection at the concentration of 100 μM. The relative activity was normalized and reported as % of inhibition. Figure S2. CD spectra of Gc48 (A), Gc56 (D), and Gc59 (G) reported as function of pH. The α-helix and β-sheet contents for peptide Gc48 (B, C), Gc56 (E, F), and Gc59 (H, I) were calculated by the server BeStSel. Figure S3. Alignment of bunyavirus fusion loops. Orthobunyaviruses showed high sequence identity (red-colored residues) [52]; on the contrary, bd, cd, and ij loops differ considerably among other genera, such as Hantavirus and Phlebovirus [51]. SBV: Schmallenberg virus; LACV: La Crosse virus; CVV: Cache Valley virus; OROV: Oropouche virus; HTNV: Hantaan virus; RVFV: Rift Valley fever virus. Table S1. Gn and Gc libraries with peptide position in M segment, names and sequences. The five active peptides are yellow-colored. [file 1600862.f1.docx]

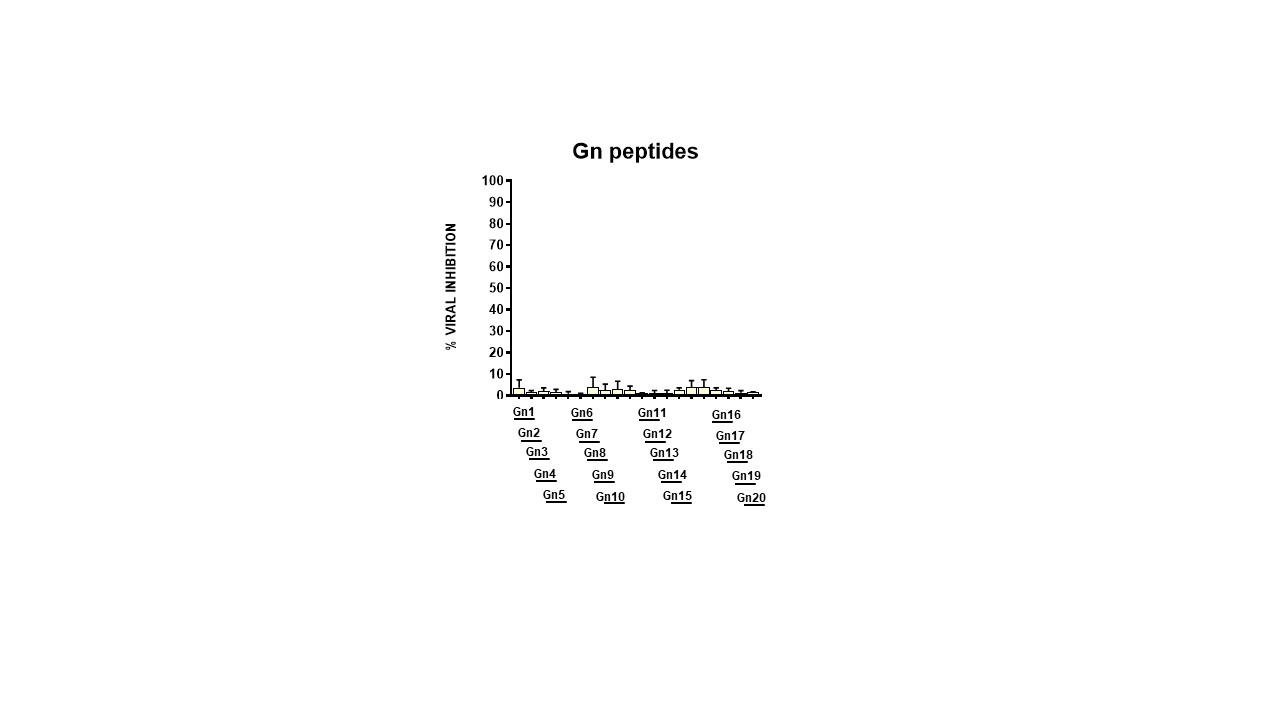


**Figure S1.** Inhibitory activity of SBV Gn peptides. Peptides were incubated simultaneously to the infection at the concentration of 100 μM. The relative activity was normalized and reported as % of inhibition.


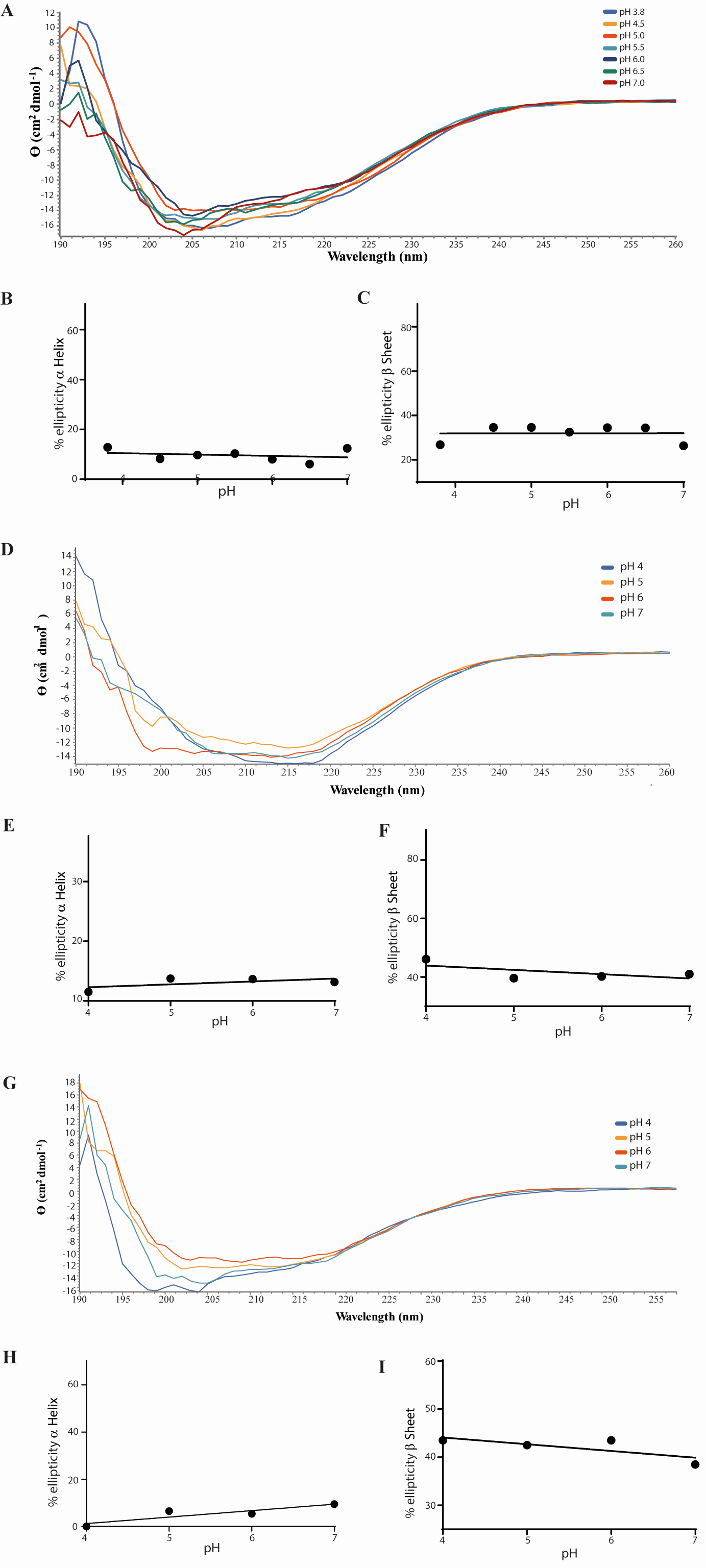


**Figure S2.** CD spectra of Gc48 (A), Gc56(D) and Gc59 (G) reported as function of pH. The α-helix and β-sheet contents for peptide Gc48 (B,C), Gc56 (E,F) and Gc59 (H,I) were calculated by the server BeStSel.


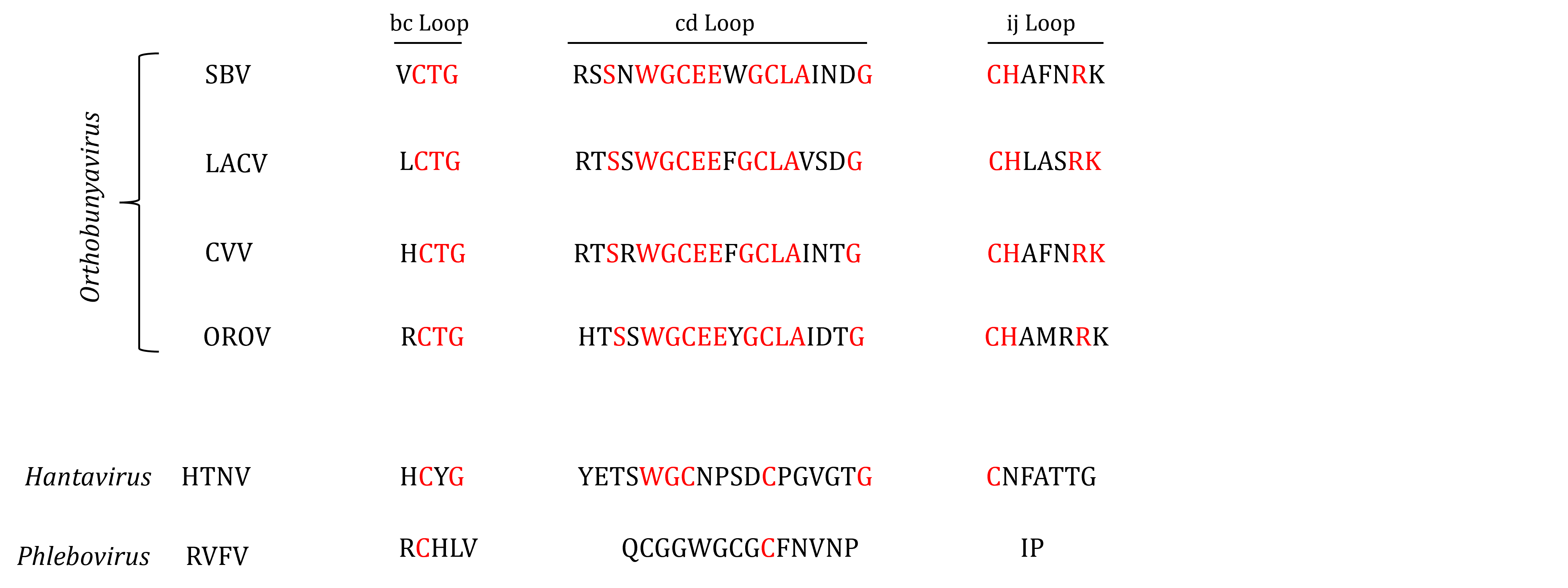


**Figure S3.** Alignment of bunyavirus fusion loops. Orthobunyaviruses showed high sequence identity (red-colored residues) [52]; on the contrary, bd, cd and ij loops differ considerably among other genera, such as *Hantavirus* and *Phlebovirus* [51]. SBV: Schmallenberg virus; LACV: La Crosse virus; CVV: Cache valley virus; OROV: Oropouche virus; HTNV: Hantaan virus; RVFV: Rift valley fever virus.

| **PEPTIDE POSITION IN M SEGMENT** | **PEPTIDE NAME** | **PEPTIDE SEQUENCE** |
| --- | --- | --- |
| M18 | Gn1 | lplkegtrgsrcflngelvktvnts |
| M33 | Gn2 | gelvktvntskvvseccvkddisii |
| M48 | Gn3 | ccvkddisiiksnaehyksgdrlaa |
| M63 | Gn4 | hyksgdrlaavikyyrlyqvkdwhs |
| M78 | Gn5 | rlyqvkdwhscnpiyddhgsfmild |
| M93 | Gn6 | ddhgsfmildidntgtlipkmhtcr |
| M108 | Gn7 | tlipkmhtcrveceialnkdtgevi |
| M123 | Gn8 | alnkdtgevilnsyrinhyrisgtm |
| M138 | Gn9 | inhyrisgtmhvsgwfknkieiple |
| M153 | Gn10 | fknkieiplentcesievtcglktl |
| M168 | Gn11 | ievtcglktlnfhacfhthksctry |
| M183 | Gn12 | fhthksctryfkgsilpelmiesfc |
| M198 | Gn13 | lpelmiesfctnlelillvtfilvg |
| M213 | Gn14 | illvtfilvgsvmmmiltktyivyv |
| M228 | Gn15 | iltktyivyvfipifypfvklyaym |
| M243 | Gn16 | ypfvklyaymynkyfklckncllav |
| M258 | Gn17 | klckncllavhpftncpstcicgmi |
| M273 | Gn18 | cpstcicgmiyttteslklhrmcnn |
| M288 | Gn19 | slklhrmcnncsgykalpktrklck |
| M303 | Gn20 | alpktrklckskisnivlcvitsli |
| M453 | Gc1 | fmlllvptivmtqetsinckniqst |
| M468 | Gc2 | sinckniqstqltiehlskcmafyq |
| M483 | Gc3 | hlskcmafyqnktsspvvineiisd |
| M498 | Gc4 | pvvineiisdasvdeqelikslnln |
| M513 | Gc5 | qelikslnlncnvidrfisessvie |
| M528 | Gc6 | rfisessvietqvyyeyiksqlcpl |
| M543 | Gc7 | eyiksqlcplqvhdiftinsasniq |
| M558 | Gc8 | ftinsasniqwkalarsftlgvcnt |
| M573 | Gc9 | rsftlgvcntnphkhicrclesmqm |
| M588 | Gc10 | icrclesmqmctstktdharemsiy |
| M603 | Gc11 | tdharemsiyydghpdrfehdmkii |
| M618 | Gc12 | drfehdmkiilnimryivpglgrvl |
| M633 | Gc13 | yivpglgrvlldqikqtkdyqalrh |
| M648 | Gc14 | qtkdyqalrhiqgklspksqsnlql |
| M663 | Gc15 | spksqsnlqlkgflefvdfilganv |
| M678 | Gc16 | fvdfilganvtiektpqtlttlsli |
| M693 | Gc17 | pqtlttlslikgahrnldqkdpgpt |
| M708 | Gc18 | nldqkdpgptpilvckspqkvvcys |
| M723 | Gc19 | kspqkvvcysprgvthpgdyiscks |
| M738 | Gc20 | hpgdyisckskmykwpslgvykhnr |
| M753 | Gc21 | pslgvykhnrdqqqacssdthclem |
| M768 | Gc22 | cssdthclemfepaertittkickv |
| M783 | Gc23 | rtittkickvsdmtysespystgip |
| M798 | Gc24 | sespystgipscnvkrfgscnvrgh |
| M813 | Gc25 | rfgscnvrghqwqiaecsnglfyyv |
| M828 | Gc26 | ecsnglfyyvsakahsktnditlyc |
| M843 | Gc27 | sktnditlyclsancldlryafrss |
| M858 | Gc28 | ldlryafrssscsdivwdtsyrnkl |
| M873 | Gc29 | vwdtsyrnkltpksinhpdienyia |
| M888 | Gc30 | nhpdienyiaalqsdiandltmhyf |
| M903 | Gc31 | iandltmhyfkplknlpaiipqykt |
| M918 | Gc32 | lpaiipqyktmtlngdkvsngirns |
| M933 | Gc33 | dkvsngirnsyieshipainglsag |
| M948 | Gc34 | ipainglsaginiampngeslfsii |
| M963 | Gc35 | pngeslfsiiiyvrrvinkasyrfl |
| M978 | Gc36 | vinkasyrflyetgptiginakhee |
| M993 | Gc37 | tiginakheevctgkcpspiphqdg |
| M1008 | Gc38 | cpspiphqdgwvtfskerssnwgce |
| M1023 | Gc39 | kerssnwgceewgclaindgclygs |
| M1038 | Gc40 | aindgclygscqdiirpeykiykks |
| M1053 | Gc41 | rpeykiykkssieqkdvevcitmah |
| M1068 | Gc42 | dvevcitmahesfcstvdvlqplis |
| M1083 | Gc43 | tvdvlqplisdriqldiqtiqmdsm |
| M1098 | Gc44 | diqtiqmdsmpniiavkngkvyvgd |
| M1113 | Gc45 | vkngkvyvgdindlgstakkcgsvq |
| M1128 | Gc46 | stakkcgsvqlysegiigsgtpkfd |
| M1143 | Gc47 | iigsgtpkfdyvchafnrkdvilrr |
| M1158 | Gc48 | fnrkdvilrrcfdnsyqscllleqd |
| M1173 | Gc49 | yqscllleqdntltiastshmevhk |
| M1188 | Gc50 | astshmevhkkvssvgtinykimlg |
| M1203 | Gc51 | gtinykimlgdfdynaystqatvti |
| M1218 | Gc52 | aystqatvtideircggcygcpegm |
| M1233 | Gc53 | ggcygcpegmacalklstntigscs |
| M1248 | Gc54 | lstntigscsiksncdtyikiiavd |
| M1263 | Gc55 | dtyikiiavdpmqseysiklncpla |
| M1278 | Gc56 | ysiklncplatetvsvsvcsasayt |
| M1293 | Gc57 | vsvcsasaytkpsisknqpkivlns |
| M1308 | Gc58 | knqpkivlnsldetsyieqhdkkcs |
| M1323 | Gc59 | yieqhdkkcstwlcrvykegisvif |
| M1338 | Gc60 | vykegisvifqplfgnlsfywrlti |
| M1353 | Gc61 | nlsfywrltiyiiislimlilflyi |
| M1368 | Gc62 | limlilflyiliplckrlkglleyn |
| M1383 | Gc63 | krlkglleynyneriyqmenkfk |

**Table S1.** Gn and Gc libraries with peptide position in M segment, names and sequences. The five active peptides are yellow-colored.
